# Effects of porn addiction on mental health and personality of nursing students: a cross-sectional study in Egypt

**DOI:** 10.1186/s12912-025-02918-z

**Published:** 2025-04-13

**Authors:** Nashwa Ahmed Hussein Abdel Karim, Mahmood Ahmed Osman, Yasmin Mohamed Mohamed Abdelmonaem, Ayman Mohamed El-Ashry

**Affiliations:** 1 Psychiatric and Mental Health Nursing, Faculty of Nursing, Zagazig University, Zagazig, Egypt; 2 Charge Nurse, Zagazig University Hospitals, Zagazig University, Zagazig, Egypt; 3 Demonstrator at Community Health Department, Faculty of Nursing, Zagazig University, Zagazig, Egypt; 4 Psychiatric and Mental Health Nursing, Faculty of Nursing, Alexandria University, Alexandria, Egypt

**Keywords:** Porn addiction, Mental health, Personality facets, Nursing students

## Abstract

**Background:**

Pornography addiction is increasingly concerning among young adults, including nursing students, who face high academic and professional pressures. Easy internet access has escalated porn consumption, leading to potential addiction with significant impacts on mental health and personality.

**Methods:**

This cross-sectional study aimed to examine the relationship between pornography addiction, mental health, and personality traits among 828 nursing students from Zagazig University and Alexandria University. Data were collected using the Pornography Addiction Screening Tool (PAST), the Depression, Anxiety, and Stress Scale (DASS-21), and the Big Five Inventory (BFI) Scale.

**Results:**

The study revealed that pornography addiction was found in 5.6% of students, with a mean addiction score of 23.07. The mean scores for the Big Five personality traits were highest for Openness (33.53) and lowest for Extraversion (23.78). The mean scores for anxiety, depression, and stress were 16.24, 15.41, and 16.82, respectively. Higher levels of pornography addiction were significantly correlated with increased anxiety (*r* = 0.369, *p* < 0.001), depression (*r* = 0.441, *p* < 0.001), and stress (*r* = 0.319, *p* < 0.001), and lower levels of personality traits except neuroticism. Regression analysis identified pornography addiction and time spent watching porn as significant predictors of both BFI and DASS-21 scores.

**Conclusion:**

The study highlights critical issues among predominantly female and single nursing students related to internet usage, pornography consumption, personality traits, and mental health. These students spend substantial time online, with a notable portion engaging in pornography viewing, which is perceived negatively. Significant levels of pornography addiction are associated with adverse effects on personality traits and increased mental health issues such as anxiety, depression, and stress. Regression analysis underscores the impact of pornography addiction and viewing duration on both personality traits and mental health outcomes.

**Nursing implications:**

Universities should implement targeted interventions to address pornography addiction among nursing students. This includes integrating awareness programs into the curriculum, providing accessible counseling services, and promoting healthy coping mechanisms for stress management. Additionally, universities should develop policies to foster a supportive academic environment and encourage responsible internet use.

**Clinical trial number:**

Not applicable.

## Introduction

Porn addiction is a complex and culturally significant issue that has not been sufficiently explored in Egypt, especially among nursing students—an essential demographic facing unique personal and professional pressures. The challenges in researching this topic arise from deeply rooted societal taboos, stigmas, and the legal implications surrounding pornography in Egypt. These barriers limit the availability of data, contributing to a significant gap in understanding porn addiction in this specific cultural context [1]. Despite these hurdles, it is crucial to examine pornography addiction among this group to grasp its real implications and create culturally sensitive interventions.

Pornography encompasses various forms of explicit content intended for sexual arousal, including images, videos, written material, and more. Anything that visually or emotionally depicts sexual activity falls into this category [2]. Worldwide, the prevalence of problematic pornography use affects a notable segment of the population, with rates varying from 3.2 to 16.6% [3]. The World Health Organization recognizes this issue as a form of Compulsive Sexual Behavior Disorder [3].

In Egypt, the influence of cultural dynamics necessitates localized research to better understand pornography consumption patterns and implications [4]. A study of over 15,000 participants found that pornography use was notably higher among men, particularly those aged 15 and younger. In contrast, higher educational levels, especially at the PhD level, correlated with decreased rates of pornography use, suggesting that education may protect against such consumption [4].

Moreover, engaging in regular physical activity appears to lessen the likelihood of pornography consumption, emphasizing the importance of lifestyle choices in addressing this issue. These insights reveal how demographic factors and personal behaviors intersect to shape pornography usage patterns, which is vital for developing effective interventions and awareness campaigns [4]. Tackling this issue in Egypt is essential for crafting tailored interventions and policies that resonate with local values and practices.

The neurobiology of pornography addiction is a subject of considerable debate in scientific and medical circles. It is essential to acknowledge that “porn addiction” is not formally classified in psychiatric manuals like the DSM-5. Nevertheless, some researchers use the term to convey patterns of compulsive and problematic consumption of pornography [5]. The persistent release of dopamine from compulsive viewing strengthens the neural pathways associated with sexual arousal, promoting a craving for more explicit content as previous stimuli become insufficient, which may lead to changes in dopamine receptors and the brain’s reward system [6].

Nursing students encounter significant academic and professional stress that can intensify mental health challenges [7]. In today’s digital era, the increased consumption of pornography among young adults, including nursing students, raises alarm bells regarding addiction and its adverse effects on mental well-being and personality traits [8]. Past research has established connections between pornography addiction and mental health issues like anxiety, depression, and stress, yet there remains a critical need to understand these relationships within the distinctive cultural framework of nursing students in Egypt. Additionally, studies indicate that personality traits, particularly neuroticism, significantly contribute to mental health issues among students engaging with internet pornography [9]. However, the intricate interconnections between pornography addiction, mental health, and personality within the conservative society of Egypt are still under-researched, particularly given the cultural taboos surrounding the subject.

Freud’s psychosexual theory offers valuable insights into the psychological aspects of pornography addiction, underscoring the significance of developmental stages and libido in influencing sexual behavior. His notion of sublimation, or redirecting sexual energy into socially acceptable forms, provides a pathway for fostering healthy psychological development and applicable interventions [10]. Freudian defense mechanisms, such as compulsive behaviors as coping strategies, may further illuminate the addictive patterns linked to pornography usage. Yet, these theories alone cannot fully encapsulate the multifaceted nature of this issue; incorporating diverse frameworks is essential to unravel the influences of cultural and environmental factors.

Contemporary approaches, including behavioral psychology, neuroscience, and addiction studies, emphasize cognitive-behavioral models and neurobiological research, which highlight the role of personality traits like impulsivity, sensation-seeking, and low self-control in predisposing individuals to addiction [11, 12]. These traits, coupled with environmental and cultural factors, create a multifaceted framework for understanding pornography addiction.

Pornography addiction is often oversimplified and misinterpreted as a moral issue rather than a complex mental health concern. Misconceptions include viewing it as a harmless aspect of modern sexuality and underestimating its impact on specific demographics. Addressing these misconceptions requires a nuanced understanding of the psychological, social, and neurobiological factors involved [13]. Individuals experiencing pornography addiction may encounter challenges related to their emotional well-being and overall mental health. Persistent consumption of explicit material can affect relationships and self-esteem and contribute to feelings of guilt, shame, or anxiety. Research suggests potential associations between pornography addiction and adverse mental health outcomes, including impaired interpersonal relationships and an increased risk of depressive symptoms [14].

This study aims to bridge the research gap by investigating the prevalence and impact of pornography addiction among nursing students in Egypt, focusing on its correlation with mental health outcomes and personality traits. By integrating Freud’s psychosexual theory with contemporary perspectives on personality and addiction, the study seeks to provide a nuanced understanding of the psychological and cultural factors driving pornography consumption in this population [10, 11]. The findings will not only contribute to the limited body of research on pornography addiction in conservative societies but also inform the development of culturally sensitive interventions and support systems for nursing students. Given their future roles as healthcare providers, nursing students must be equipped to address mental health issues, both in themselves and their patients. This study underscores the importance of fostering awareness and empathy within the nursing profession, ensuring that future healthcare providers are prepared to navigate the complexities of mental health in a rapidly evolving digital landscape.

Given the sensitive nature of the topic and the cultural context in Egypt, a cross-sectional design was deemed appropriate for this study. This design allows for the collection of data from a large sample of nursing students at a single point in time, providing a snapshot of the prevalence of pornography addiction and its associated mental health and personality traits. The cross-sectional approach is particularly suitable for exploring correlations and identifying potential predictors, such as time spent watching pornography, without the need for long-term follow-up, which may be challenging in a culturally conservative setting [15].

### Objective if the study

Assess the effect of pornography addiction on mental health and personality among nursing students.

### Research question

How pornography addiction affects mental health and personality among nursing students?

### Design

A cross-sectional design was conducted at all academic levels at the nursing faculties of Zagazig and Alexandria Universities in Egypt.

### Setting

The study was conducted at the nursing faculties of Zagazig and Alexandria Universities in Egypt, offering extensive academic programs and modern facilities for nursing education. These faculties provide theoretical and practical training through well-equipped lecture halls and cater to a significant student population across various nursing specialties.

### Sample size calculation and sampling

The study calculated a minimum sample size of 629 participants using the Epi-Info program, considering a population of 12,000 nursing students, an expected frequency of 50%, an acceptable error of 10%, a design effect of 1, a confidence coefficient of 99%, and a power of 80%. To account for potential attrition bias and non-response rates, the sample size was increased to 828, ensuring the final analyzed sample would maintain statistical power and confidence. This adjustment is crucial in cross-sectional studies, where dropout and incomplete responses are common. Additionally, the larger sample size helps mitigate the inherent limitations of convenience sampling by improving the likelihood of capturing a diverse and representative subset of the population, including varying demographics such as age, gender, and university affiliation. While convenience sampling may introduce selection bias, the increased sample size enhances the study’s robustness and external validity.

### Recruitment process

**Fig. 1:**
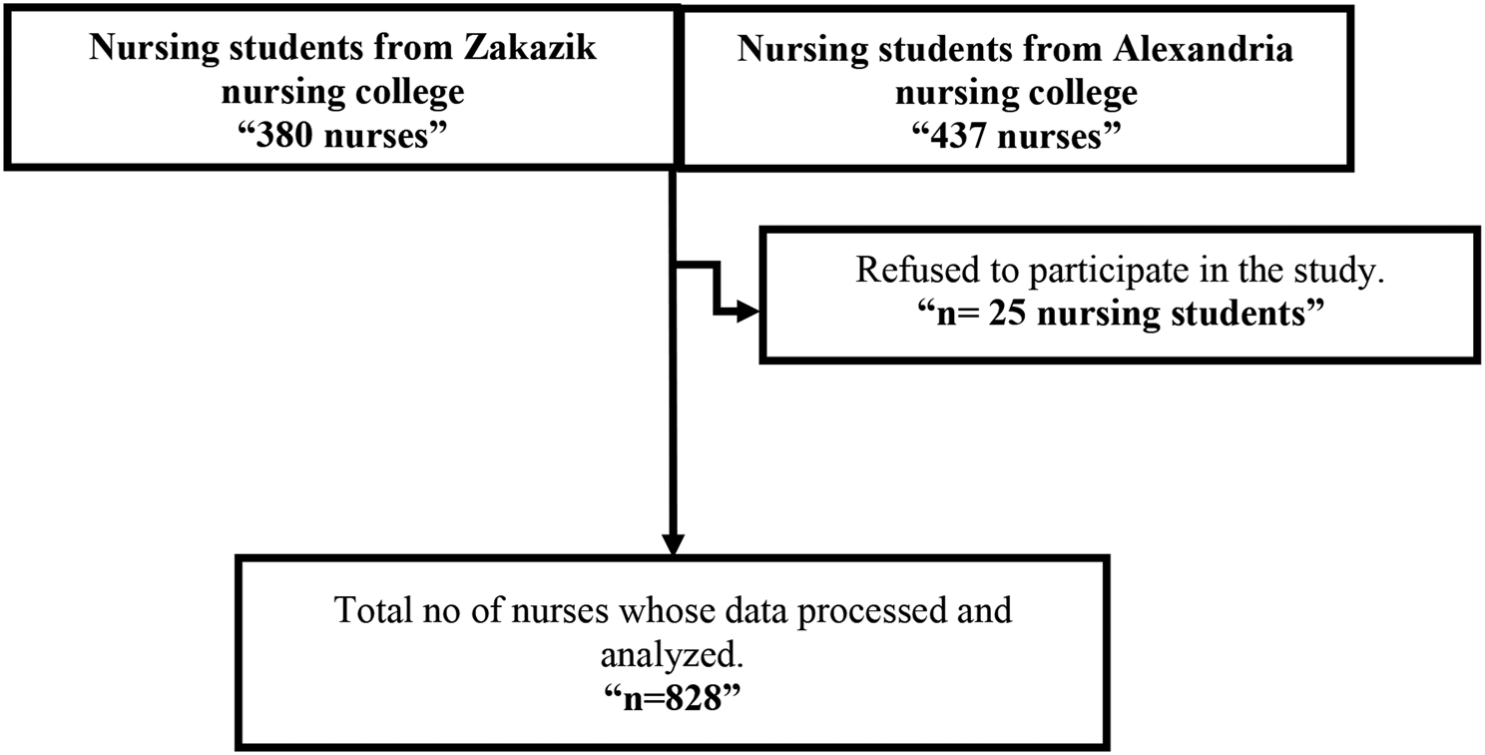
Flow chart of participants’ recruitment process

The research was conducted over three months, starting in early January 2024 and concluding at the end of March 2024. The total number of enrolled students was 380 for the nursing college at Zakazik University and 473 for the college of nursing at Alexandria University. However, 25 students (15 from Zakazik and 10 from Alexandria College of Nursing) were excluded from the study as they refused to participate (Fig. 1 flow chart of the recruitment process).

### Tools for data collection

The researchers employed a questionnaire consisting of four sections for gathering data:

### Section I: demographic data sheet

Participants were asked to provide information on their age, gender, marital status, and academic year. Additionally, participants were queried about their awareness of resources related to porn addiction, major stressors they experienced, and their internet usage habits. Further inquiries included the age at which they were first exposed to pornography, the average time spent on pornographic content weekly, and the types of pornography consumed. Participants also assessed the perceived impact of pornography on their well-being and indicated whether they had attempted to reduce or stop porn consumption. Finally, participants identified primary triggers leading to porn consumption.

### Section II: pornography addiction screening tool (PAST)

The tool developed by Bulkley (2013) is designed to assess porn addiction in college students, featuring 25 items scored on a 5-point scale ranging from 0 (Never) to 4 (Frequently) [16]. Based on the total score, individuals fall into one of four categories that guide intervention recommendations. Scores between 0 and 25 indicate normal sexual development and curiosity; no treatment is needed, though monitoring online activity and providing education on the risks of pornography, as well as fostering open discussions about sexuality, are recommended. A score of 26 to 49 raises concerns about involvement with pornography. In this range, individuals may be vulnerable to future addiction, warranting monitoring, preventative education, and open discussions about sexuality. Scores between 50 and 69 suggest an emerging addiction, indicating a need for vigilant monitoring and treatment to prevent further addiction. Individuals in this range may display distorted views on sexuality, hypersexual behaviors, and stagnating healthy coping skills, with an elevated risk of emotional, social, and legal issues related to pornography addiction. Finally, scores from 70 to 100 indicate a total addiction, necessitating vigilant monitoring and professional treatment, as individuals in this category typically cannot break the addiction without support. At this level, addiction tends to replace healthy coping skills, distorting judgment and priorities and leading to high risks of emotional, social, and legal complications. This structured assessment approach helps professionals gauge the severity of porn addiction and recommend appropriate interventions based on the degree of addiction. The internal consistency in the current study was assessed using Cronbach’s Alpha. The Cronbach’s alpha value was 0.91.

### Section III: Arabic version of depression, anxiety, and stress scale (DASS 21)

This tool was developed by Lovibond Lovibond in 1995 to assess levels of depression, anxiety, and stress [17] and validated in Arabic by Ali et al., 2017 [18]. This self-report instrument comprises three scales, each containing 7 items, designed to measure emotional states related to depression, anxiety, and stress. Participants rate their responses on a 4-point scale, ranging from “0 = did not apply to me at all” to “3 = applied to me very much.” The depression scale evaluates feelings of dysphoria, hopelessness, devaluation of life, self-deprecation, lack of interest or involvement, anhedonia, and inertia. The anxiety scale focuses on autonomic arousal, skeletal muscle effects, situational anxiety, and the subjective experience of anxious affect. Lastly, the stress scale assesses difficulty relaxing, nervous arousal, and tendencies towards being easily upset, agitated, irritable, or over-reactive.

The scoring system for the DASS21 measures depression, anxiety, and stress by summing scores from relevant items in each category. To interpret scores based on conventional severity labels, results are classified as follows: for depression, scores range from 0 to 9 (normal), 10–13 (mild), 14–20 (moderate), 21–27 (severe), and 28+ (extremely severe); for anxiety, scores are 0–7 (normal), 8–9 (mild), 10–14 (moderate), 15–19 (severe), and 20+ (extremely severe); and for stress, scores range from 0 to 14 (normal), 15–18 (mild), 19–25 (moderate), 26–33 (severe), and 34+ (extremely severe). It’s important to note that scores on the DASS21 must be multiplied by 2 to determine the final score for accurate interpretation. The internal consistency in the current study was assessed using Cronbach’s Alpha. The Cronbach’s alpha value was 0.73.

### Section V: the Arabic version of big five inventory (BFI)

John and Srivastava (1999) designed the tool to measure personality facets [19], and it was translated and validated into Arabic by Alansari (2016) [20]. The scale consisted of 44 items, and Responses were measured on a 5-point scale (1 = disagree strongly, 2 = disagree a little, 3 = neither agree nor disagree, 4 = agree a little, 5 = agree strongly). The BFI (Big Five Inventory) scale scoring system categorizes items to measure five personality traits. For Extraversion, 8 items are included. Agreeableness is assessed through 9 items. For Conscientiousness, 9 items were included. Neuroticism is measured by 8 items. Finally, Openness is scored based on 10 items. Reverse-scored items are calculated by adjusting the scores to reflect the inverse of their original rating. The internal consistency in the current study was assessed using Cronbach’s Alpha. The Cronbach’s alpha value was 0.82.

### Procedure

#### Ethical considerations

The research adhered to stringent ethical standards to protect participants’ rights, dignity, and confidentiality. Ethical approval was obtained from the Research Ethics Committee (REC) at Zakazik University College of Nursing (**Code: Zu.Nur.REC#061/11/2023**), and additional permissions were secured from the relevant ethics committees at each participating nursing college. The vice deans of the involved universities provided written support, underscoring the institutional commitment to ethical research practices. All participants were provided with detailed information about the study’s purpose, procedures, potential risks, and benefits, and written informed consent was obtained to ensure voluntary participation. Given the sensitive nature of the research topic, strict measures were implemented to safeguard confidentiality and anonymity. Data were collected using anonymized identifiers, and participants were assigned unique codes to replace any personally identifiable information. All data were stored in password-protected electronic files and locked physical cabinets, with access restricted to the core research team. Only aggregated and de-identified data were used during analysis, and individual responses were not disclosed in any reports or publications. Participants were informed of their right to withdraw at any stage, and if they chose to do so, their data were promptly removed and destroyed. The research team underwent training on ethical practices, particularly in handling sensitive data, and maintained transparency with participants about data usage and protection. These measures ensured that privacy and confidentiality were rigorously maintained, fostering trust and upholding the integrity of the research.

#### Validity and pilot study

The validity of the Pornography Addiction Screening Tool (PAST) for use among Egyptian nursing students was rigorously established through a comprehensive cultural adaptation and validation process. The tools were first translated into Arabic by bilingual experts fluent in English and Arabic to ensure linguistic accuracy and semantic equivalence. This was followed by independent translators’ back-translation into English to identify and resolve any discrepancies between the original and translated versions. A panel of five experts, including psychologists, linguists, and nursing educators with expertise in Egyptian culture, evaluated the translated tools for artistic relevance, clarity, and appropriateness. Their review focused on ensuring the items aligned with the conservative and religious values prevalent in Egyptian and Egyptian nursing students’ specific experiences and expressions. The Content Validity Ratio (CVR) was calculated to quantify the level of agreement among the experts, with values exceeding the critical threshold of 0.99, indicating substantial agreement on the relevance of each item.

Additionally, the Content Validity Index (CVI) was computed, yielding values above 0.80, further confirming the cultural and conceptual appropriateness of the tools. Pilot testing was conducted with a sample of 120 Egyptian nursing students to refine the tools further. This step ensured that the items were culturally sensitive, easily comprehensible, and free from ambiguity. However, no changes were needed as the instruments were considered appropriate, understandable, and practical.

#### Data collection

Once permission was granted to proceed with the study, the researchers obtained the population size for all the studied samples. The researcher then met with the vice deans for students’ affairs and education in the nursing faculty and explained the study aims, procedures, and data collection forms, including obtaining official permission to conduct the study and access the study sample.

The researchers carried out a face-to-face interview in solitary to assess study variables. The studied sample was asked to read each sentence carefully and slowly. The pilot study revealed that the average time to fill in all the tools was 20–30 min.

#### Bias mitigation

Several measures were implemented to ensure the reliability and validity of the responses and address potential biases during the data collection process. First, the study emphasized **anonymity and confidentiality** to encourage honest reporting. Participants were assured that their responses would remain private and not be linked to their identities or academic records. This approach was critical in a culturally conservative context like Egypt, where sensitive topics such as pornography addiction may carry stigma. Second, the research team provided **clear instructions** and was available to clarify participants’ questions or concerns about the survey instruments. This ensured that participants fully understood the items and could respond accurately.

Additionally, the survey was administered in a controlled environment, free from external distractions, to minimize errors and enhance focus. To mitigate **social desirability bias**, the study avoided leading questions and used standardized, validated tools (e.g., PAST, DASS-21, and BFI) widely recognized for their reliability in assessing sensitive behaviors and psychological states. Participants were also informed that there were no right or wrong answers, further reducing the pressure to respond in a socially acceptable manner.

After data collection, the researcher and a specialist in statistics carried out all necessary steps to check the completeness of the data and proceeded to score the members’ answers. The data was collected over three months from the beginning of April to the end of July, 2024.

### Statistical analysis of the data

The data were analyzed using IBM SPSS software, version 23.0. Qualitative variables were presented as numbers and percentages, while quantitative data were expressed as means and standard deviations. Statistical significance was evaluated at the 5% level. A variety of statistical tests were applied to the analysis. The Student t-test was used to compare two groups when data were normally distributed, while the F-test (ANOVA) facilitated comparisons among more than two groups for normally distributed data. The Pearson correlation coefficient assessed correlations between two normally distributed quantitative variables. The data subjected to linear regression analysis were conducted to identify the most significant independent factors affecting BFI and DASS21 outcomes. The data was assessed for normality to ensure the validity of the statistical assumptions. The Shapiro-Wilk test was used to evaluate the data distribution, and the results indicated that the data followed a normal distribution (*p* > 0.05). This confirms that the assumptions of linear regression were met, allowing for robust and reliable analysis. The findings derived from the linear regression models are statistically valid and can be interpreted confidently.

## Results

Table 1 found that the average age of the nursing students was 20.74 years (SD: 1.70), with 62.1% being younger than 20 and 37.9% aged 20 or older. The sample was predominantly female (63.0%), and the majority were single (92.9%). Academically, 39.3% were in their fourth year, 31.6% in their third year, 22.2% in their first year, and 6.9% in their second year. On average, students used the internet for 15.94 h daily (SD: 2.70), with 34.2% using it for more than 4 h daily. Regarding pornography viewing, 55.5% watched for less than 1 h per week, while 2.7% watched for more than 5 h weekly. Most students (60.9%) perceived pornography as having adverse effects, with boredom (54.3%) being the primary motive for viewing.

**Table 1:** Relation between socio-demographic characteristics of the nursing students’ and study variables (*n* = 828)

	No	%
**Age**	**20.74 ± 1.70**	
≤ 20	314	37.9
> 20	514	62.1
**Sex**		
Male	306	37.0
Female	522	63.0
**Marital status**		
Single	769	92.9
Married	59	7.1
**Academic level**		
1st	184	22.2
2nd	57	6.9
3rd	262	31.6
4th	325	39.3
**Average time using internet per day**		
Less than 2 h	190	22.9
2–4 h	355	42.9
More than 4 h	283	34.2
	**15.94 ± 2.70**	
**Average time watching porn sites per week**		
Less than 1 h.	460	55.5
1–3 h.	231	27.9
3–5 h.	115	13.9
5+	22	2.7
**Effect of porn movies from students perspective**		
Negative	505	60.9
No effect	173	20.9
Positive	150	18.2
**Motives to watch porn**		
Feeling bored	450	54.3
Curiosity	153	18.4
Lack of confidence	75	9.2
Romantic movies and series	150	18.1

Table 2 revealed that the mean score for pornography addiction was 23.07 (SD: 26.05), with 63.3% in the “normal” category, 15.5% showing concern, 15.7% needing treatment, and 5.6% classified as addicts. The Big Five Inventory personality traits ranked by mean scores were Openness (33.53), Agreeableness (31.63), Conscientiousness (29.20), Neuroticism (25.66), and Extraversion (23.78). Also, assessment using the DASS-21 among nursing students revealed mean scores of 16.24 (SD: 9.87) for anxiety, 15.41 (SD: 10.67) for depression, and 16.82 (SD: 10.44) for stress. For anxiety, 20.9% were “normal” and 36.8% “extremely severe.” For depression, 32.5% were “normal,” and 16.7% “extremely severe.” For stress, 47.6% were “normal,” 15.3% “mild,” 16.2% “moderate,” 12.1% “severe,” and 8.8% “extremely severe.” The overall mean score for the DASS-21 was 48.47 (SD: 29.02), indicating varying levels of psychological distress among the participants.

**Table 2:** Distribution of score for the study variables (*n* = 828)

	Mean	± SD
	No.	%
**pornography addiction screening tool**	**23.07**	**26.05**
Normal (0–25)	524	63.3
Reason for concern (26–49)	128	15.5
Emerging (50–69)	130	15.7
Addict individual (70–100)	46	5.6
**The big five inventory of personality**	**Mean**	**± SD**
Extraversion	23.78	3.60
Agreeableness	31.63	5.47
Conscientiousness	29.20	4.88
Neuroticism	25.66	5.07
Openness	33.53	5.93
**Overall The big five inventory**	**143.79**	**14.43**
**depression anxiety stress scale**		
**Anxiety**	**16.24**	**9.87**
Normal (0–9)	173	20.9
Mild (10–13 )	34	4.1
Moderate (14–20)	178	21.5
Severe (21–27)	138	16.7
Extremely Severe (28+)	305	36.8
**Depression**	**15.41**	**10.67**
Normal (0–7)	269	32.5
Mild (8–9)	76	9.2
Moderate (10–14)	255	30.8
Severe (15–19)	90	10.9
Extremely Severe (20+)	138	16.7
**Stress**	**16.82**	**10.44**
Normal (0–14)	394	47.6
Mild (15–18)	127	15.3
Moderate (19–25)	134	16.2
Severe (26–33)	100	12.1
Extremely Severe (34+)	73	8.8
**Overall DASS21**	**48.47**	**29.02**

Table 3 reveals significant relationships between pornography addiction (PAST), personality traits, and mental health outcomes, which align closely with the study’s objectives of understanding the psychological and emotional factors associated with addiction. The negative correlations between PAST and traits like **Extraversion (r = -0.259, p < 0.001), Agreeableness (r = -0.396, p < 0.001)**, and **Conscientiousness (r = -0.354, p < 0.001)** suggest that individuals with pornography addiction often struggle with sociability, cooperation, and self-discipline, highlighting the need for interventions that address these personality dimensions. Additionally, the positive correlation between PAST and **Neuroticism (r = 0.254, p < 0.001)** underscores the role of emotional instability in addiction, emphasizing the importance of incorporating emotional regulation strategies into treatment programs. While unexpected, the strong association between PAST and **Openness (r  = 0.560, p < 0.001)** points to the need for targeted educational efforts to redirect curiosity and openness toward healthier activities. Furthermore, the positive correlations between PAST and mental health issues such as **Anxiety (r = 0.369, p < 0.001), Depression (r = 0.441, p < 0.001)**, and **Stress (r = 0.319, p < 0.001)** reinforce the study’s objective of exploring the mental health burden associated with addiction, suggesting that integrated mental health support should be a core component of addiction interventions.

**Table 3:** Correlation between the study variables (*n* = 828)

		PAST	Extraversion	Agreeableness	Conscientiousness	Neuroticism	Openness	Overall BFI	Anxiety	Depression	Stress
Extraversion	r	-0.259*									
	p	< 0.001*									
Agreeableness	r	-0.396*	0.060								
	p	< 0.001*	0.087								
Conscientiousness	r	-0.354*	0.243*	0.369*							
	p	< 0.001*	< 0.001*	< 0.001*							
Neuroticism	r	0.254*	0.134*	0.052	0.174*						
	p	< 0.001*	< 0.001*	0.134	< 0.001*						
Openness	r	0.560*	0.301*	0.356*	0.227*	0.189*					
	p	< 0.001*	< 0.001*	< 0.001*	< 0.001*	< 0.001*					
Overall BFI	r	-0.654*	0.431*	0.684*	0.571*	0.356*	0.765*				
	p	< 0.001*	< 0.001*	< 0.001*	< 0.001*	< 0.001*	< 0.001*				
Anxiety	r	0.369*	-0.190*	-0.277*	-0.259*	0.147*	-0.360*	-0.440*			
	p	< 0.001*	< 0.001*	< 0.001*	< 0.001*	< 0.001*	< 0.001*	< 0.001*			
Depression	r	0.441*	-0.195*	-0.293*	-0.273*	0.163*	-0.397*	-0.473*	0.796*		
	p	< 0.001*	< 0.001*	< 0.001*	< 0.001*	< 0.001*	< 0.001*	< 0.001*	< 0.001*		
Stress	r	0.319*	-0.169*	-0.237*	-0.193*	0.114*	-0.301*	-0.362*	0.818*	0.831*	
	p	< 0.001*	< 0.001*	< 0.001*	< 0.001*	< 0.001*	< 0.001*	< 0.001*	< 0.001*	< 0.001*	
Overall DASS21	r	0.403*	-0.197*	-0.287*	-0.258*	0.151*	-0.377*	-0.454*	0.927*	0.938*	0.944*
	p	< 0.001*	< 0.001*	< 0.001*	< 0.001*	< 0.001*	< 0.001*	< 0.001*	< 0.001*	< 0.001*	< 0.001*

PAST: Pornography addiction screening tool BFI: big five inventory r: Pearson coefficient *: Statistically significant at *p* ≤ 0.05

Table 4 shows males had significantly lower BFI scores (140.79) than females (145.55) (*p* < 0.001), with no significant difference in DASS-21 scores by gender. Second-year students had the lowest BFI score (136.95) and highest DASS-21 score (59.40) (BFI: *p* < 0.001; DASS-21: *p* = 0.022). Students using the internet less than 2 h daily had the lowest BFI score (138.30) and highest DASS-21 score (62.20), while those using it 2–4 h daily scored highest on BFI (145.66) and lowest on DASS-21 (46.13) (BFI: *p* = 0.001; DASS-21: *p* = 0.001). Students watching porn for more than 5 h weekly had the lowest BFI score (134) and highest DASS-21 score (59.55) (BFI: *p* < 0.001; DASS-21: *p* = 0.047). Those perceiving no effect of porn had the lowest BFI score (139.05) and highest DASS-21 score (57.52) (BFI: *p* < 0.001; DASS-21: *p* = 0.002). Motives for watching porn showed significant differences in DASS-21 scores for lack of confidence (*p* = 0.003) and romantic movies (*p* = 0.003) but not for BFI scores.

**Table 4:** Relation between socio-demographic characteristics of the nursing students’ and study variables (*n* = 828)

	BFI		DASS21	
	Mean ± SD	Test of sig.	Mean ± SD	Test of sig.
**Age**				
≤ 20	143.20 ± 13.53	t = 0.948	50.42 ± 29.63	t = 1.512
> 20	144.15 ± 14.95	*P* = 0.344	47.28 ± 28.60	*P* = 0.131
**Sex**				
Male	140.79 ± 12.74	t = 4.850*	50.15 ± 27.99	t = 1.275
Female	145.55 ± 15.06	*P* < 0.001*	47.49 ± 29.59	*P* = 0.203
**Marital status**				
Single	143.84 ± 14.38	t = 0.334	48.47 ± 28.73	t = 0.010
Married	143.19 ± 15.14	*P* = 0.739	48.51 ± 32.84	*P* = 0.992
**Academic level**				
1st	141.57 ± 13.04		49.45 ± 29.20	
2nd	136.95 ± 14.06	F = 7.798*	59.40 ± 29.39	F = 3.239*
3rd	144.72 ± 13.86	*P* < 0.001*	46.85 ± 29.26	*P* = 0.022*
4th	145.50 ± 15.22		47.31 ± 28.35	
**Average time using internet per day**				
Less than 2 h	138.30 ± 19.29		62.20 ± 37.07	
2–4 h	145.66 ± 14.43	F = 5.893*	46.13 ± 27.41	F = 5.952*
More than 4 h	143.63 ± 13.39	*P* = 0.001*	46.83 ± 28.54	*P* = 0.001*
**Average time watching porn sites per week**				
Less than 1 h.	142.40 ± 13.92		50.30 ± 29.21	
1–3 h.	140.06 ± 8.78		46.04 ± 22.76	
3–5 h.	144.00 ± 9.19	F = 9.957*	48.40 ± 20.65	F = 2.423*
5+	134.00 ± 12.82	*P* < 0.001*	59.55 ± 25.17	*P* = 0.047*
**Effect of porn movies from students perspective**				
Negative	142.98 ± 13.27		48.70 ± 26.49	
No effect	139.05 ± 14.37	F = 13.302*	57.52 ± 33.15	F = 4.873*
Positive	139.0 ± 12.04	*P* < 0.001*	47.28 ± 30.32	*P* = 0.002*
**Motives to watch porn**				
Feeling bored	142.48 ± 13.74	t = 1.438 *P* = 0.151	49.59 ± 29.53	t = 0.761 *P* = 0.447
Curiosity	143.64 ± 14.91	t = 1.065 *P* = 0.290	53.83 ± 26.52	t = 1.140 *P* = 0.255
Lack of confidence	141.22 ± 11.75	t = 0.886 *P* = 0.376	45.11 ± 26.68	t = 3.029* *P* = 0.003*
Romantic movies and series	142.0 ± 3.46	t = 0.090 *P* = 0.930	28.80 ± 17.62	t = 3.824* *P* = 0.003*

F: One way ANOVA test t: Student t-test U: Mann Whitney test H: H for Kruskal Wallis test

*: Statistically significant at *p* ≤ 0.05

**In** Table 5, pornography addiction (PAST) was a significant negative predictor of BFI scores (B = -0.362, Beta = -0.654, *p* < 0.001), explaining 42.8% of the variance. Adding average time spent watching porn per week in Step 2 further negatively impacted BFI scores (B = -2.136, Beta = -6.781, *p* < 0.001), alongside PAST (B = -0.354, Beta = -0.639, *p* < 0.001), increasing the explained variance to 45.3%. In Step 3, gender (female) emerged as a significant positive predictor (B = 2.448, Beta = 0.082, *p* = 0.003), alongside PAST (B = -0.350, Beta = -0.632, *p* < 0.001) and average time watching porn (B = -1.414, Beta = -4.488, *p* = 0.014). This final model explained 46.5% of the variance in BFI scores, highlighting the combined influence of pornography addiction, viewing habits, and gender on personality traits.

**Table 5:** Stepwise linear regression analysis showing factors affect BFI (*n* = 828)

Variable	B	Beta	t	*p*	95% CI	
					LL	UL
**Step1**						
PAST	-0.362	-0.654	-24.853*	< 0.001*	-0.391	-0.334
R^2^=,0.428 Adjusted R^2^ = 0.427,,F = 617.667^*^,*p* < 0.001^*^						
**Step2**						
PAST	-0.354	-0.639	-24.676*	< 0.001*	-0.382	-0.326
Average time watching porn sites per week	-2.136	-6.781	-3.848*	< 0.001*	-3.225	-1.046
R^2^ = 0.453, Adjusted R^2^ = 0.451,,F = 227.802^*^,*p* < 0.001^*^						
**Step3**						
PAST	-0.350	-0.632	-24.610*	< 0.001*	-0.378	-0.322
Average time watching porn sites per week	-1.414	-4.488	-2.454*	0.014*	-2.544	-0.283
Sex (Female)	2.448	0.082	2.974*	0.003*	0.832	4.064
R^2^ = 0.465, Adjusted R^2^ = 0.462,,F = 143.110^*^,*p* < 0.001^*^						

F, p: f and p values for the model; R^2^: Coefficient of determination; B: Unstandardized Coefficients

Beta: Standardized Coefficients; t: t-test of significance; LL: Lower limit UL: Upper Limit

*: Statistically significant at *p* ≤ 0.05

Table 6 shows that pornography addiction (PAST) emerged as a significant positive predictor of DASS-21 scores (B = 0.448, Beta = 0.403, *p* < 0.001), explaining 16.2% of the variance. Adding average time spent watching porn sites per week in Step 2 revealed a significant negative impact on DASS-21 scores (B = -5.098, Beta = -0.077, *p* = 0.016), while PAST remained a strong positive predictor (B = 0.442, Beta = 0.397, *p* < 0.001). This model slightly improved the explained variance to 16.8% (R² = 0.168, Adjusted R² = 0.166, *p* < 0.001).

**Table 6:** Stepwise linear regression analysis showing factors affect DASS21 (*n* = 828)

Variable	B	Beta	t	*p*	95% CI	
					LL	UL
**Step1**						
PAST	0.448	0.403	12.638*	< 0.001*	0.379	0.518
**R**^**2**^** = 0.162, Adjusted R**^**2**^ = 0.161,,F = 159.712^*^,p < 0.001^*^						
**Step2**						
PAST	0.442	0.397	12.480*	< 0.001*	0.373	0.512
Average time watching porn sites per week	-5.098	-0.077	-2.421*	0.016*	-9.231	-0.965
**R**^**2**^** = 0.168, Adjusted R**^**2**^ = 0.166,,F = 83.257^*^,p < 0.001^*^						

F, p: f and p values for the model; R^2^: Coefficient of determination; B: Unstandardized Coefficients

Beta: Standardized Coefficients; t: t-test of significance; LL: Lower limit UL: Upper Limit

*: Statistically significant at *p* ≤ 0.05

## Discussion

The findings of this study highlight the interplay between pornography addiction, personality traits, and mental health among nursing students, emphasizing the critical role of cultural and religious factors in shaping behaviors.

The relatively low levels of reported pornography addiction in the current study can be attributed to Egypt’s strong cultural and religious norms, particularly Islamic teachings that emphasize modesty and discourage sexually explicit content [21, 22]. However, social desirability bias may contribute to underreporting, as discussing pornography remains taboo in conservative societies. Additionally, the demanding nature of nursing education, which involves rigorous coursework, clinical responsibilities, and ethical obligations, likely reduces opportunities for excessive engagement with pornography, a finding consistent with research on structured academic environments limiting compulsive behaviors [22, 23].

Beyond pornography use, the study findings underscores the significant mental health challenges faced by nursing students with high levels of anxiety, depression, and stress. These psychological burdens stem from academic pressures, clinical workload, and social expectations, aligning with previous studies on stressors in medical education [24, 25]. To address these concerns, resilience-building interventions are crucial. Mindfulness-Based Stress Reduction (MBSR) techniques, such as meditation and breathing exercises, can help students develop emotional regulation and manage stress effectively. Cognitive-behavioral therapy (CBT) workshops provide students with coping strategies to shift negative thinking patterns and improve problem-solving skills [26, 27].

Furthermore, peer support and mentorship programs, particularly relevant in collectivist societies like Egypt, can foster a sense of belonging and provide emotional support, reducing feelings of isolation and stress [28]. Since religious coping mechanisms are central to Egyptian culture, integrating faith-based counseling and Islamic therapeutic approaches into university mental health programs can enhance students’ resilience [29, 30]. Physical activity interventions, such as structured fitness programs, yoga sessions, or university-led sports initiatives, can further support mental well-being by reducing stress and promoting overall psychological health [31].

The relationship between pornography addiction and personality traits suggests that individuals with higher addiction tendencies exhibit lower levels of Conscientiousness, Extraversion, and Agreeableness, which are crucial for self-regulation and social functioning [32, 40]. In Egyptian society, where self-discipline and family values are strongly emphasized, individuals with higher conscientiousness may be more resistant to addictive behaviors due to their goal-oriented nature and adherence to moral principles [33].

Additionally, the significant correlation between pornography addiction and Neuroticism suggests that individuals with emotional instability may use pornography as a coping mechanism for stress and anxiety [29, 40]. In a cultural context where emotional expression is often restrained due to societal expectations, this may lead to unhealthy coping strategies, reinforcing the need for alternative emotional support systems such as psychological counseling and stress-management programs [34].

The unexpected correlation between pornography addiction and Openness may reflect a broader trend of curiosity influenced by globalized media exposure. As internet accessibility increases, younger generations in Egypt are exposed to diverse content, including explicit material, creating a conflict between cultural restrictions and personal curiosity [30].

Educational interventions should focus on guiding this curiosity towards positive, constructive learning experiences, such as digital literacy programs that educate students on responsible internet use [35]. Interestingly, moderate internet usage (2–4 h daily) was associated with better personality traits and lower levels of depression, anxiety, and stress, suggesting that structured and purposeful online engagement may act as a protective factor rather than a risk factor for mental health issues [35]. Excessive internet use, particularly for passive activities like pornography consumption, may exacerbate stress and anxiety, reinforcing the importance of digital literacy education in universities [36, 37].

Furthermore, gender differences in personality traits and addiction patterns reflect societal expectations. Males scored lower in Agreeableness and Conscientiousness, potentially due to gender roles that promote assertiveness and independence over cooperation and self-discipline [33]. However, both males and females reported similar levels of stress, depression, and anxiety, indicating that the mental health burden is shared across genders [34].

The significant impact of academic level on mental health suggests that second-year students experience higher stress and lower personality scores, likely due to increased academic workload and clinical exposure [34]. The findings also highlight that pornography addiction significantly predicts mental health outcomes, explaining 16.2% of the variance in depression, anxiety, and stress [37].

The negative association between moderate pornography use and DASS-21 scores suggests a potential threshold effect, where controlled use does not necessarily worsen mental health outcomes, but excessive use does [38, 39]. This may indicate that some students use pornography as a temporary coping mechanism for stress, highlighting the need for alternative strategies that promote healthier coping behaviors [37].

### Strengths and limitations of the study

The study has several notable strengths that enhance its contribution to the field. First, it addresses a highly sensitive and under-researched topic—pornography addiction—within the conservative cultural context of Egypt, breaking the silence surrounding this issue among nursing students. This is particularly significant given the societal taboos and stigma associated with pornography in the region. Second, the study employs a robust methodological design, utilizing validated tools such as the PAST, the DASS-21, and the BFI to ensure reliable and comprehensive data collection. The large sample size of 828 participants further strengthens the study’s statistical power and generalizability. The study highlights the unique pressures nursing students face, making the findings highly relevant to this critical demographic. By examining the interplay between pornography addiction, personality traits, and mental health, the study provides valuable insights that can inform culturally sensitive interventions.

The study on pornography consumption in Egypt reveals several limitations that may impact the validity and generalizability of its findings. One significant challenge is the topic’s culturally sensitive nature, which may have led to underreporting or biased self-reports due to societal stigma. Despite implementing rigorous measures to mitigate social desirability bias—such as ensuring anonymity and confidentiality and utilizing standardized, validated tools—the conservative cultural context may still have influenced participants’ responses.

Additionally, the cross-sectional design restricts causal inferences, while the predominance of female participants (63%) raises concerns about generalizability, particularly regarding gender-specific behaviors. Conducting the study within only two universities further limits its geographic scope, and reliance on face-to-face interviews introduces the possibility of interviewer bias. Moreover, there is a need for a more nuanced cultural adaptation of psychometric tools and consideration of external influences, such as family or peer pressures. The relatively small percentage of participants classified as addicted to pornography (5.6%) and the lack of distinction in internet usage activities may also diminish the robustness of the conclusions.

The study’s findings may be further complicated by gender-specific biases in data collection. Cultural norms in Egypt often impose more significant restrictions on women’s expressions of sexuality, which could result in underreporting or more cautious responses among female participants. In contrast, while male participants might be more willing to discuss pornography use, they could also experience social desirability bias, as acknowledging such behavior may be perceived as culturally inappropriate. Although the study did not explicitly analyze gender differences in response rates or participation challenges, these dynamics remain a potential limitation that could affect the generalizability of the findings.

## Conclusion and recommendations

This study highlights significant findings on the interplay between pornography addiction, personality traits, and mental health among nursing students. A concerning minority exhibited high levels of pornography addiction, as measured by the Problematic Pornography Use Scale (PAST), which was linked to specific personality traits assessed by the Big Five Inventory (BFI). Higher addiction levels correlated with lower extraversion, agreeableness, openness, and conscientiousness but higher neuroticism. These traits, in turn, influenced mental health outcomes, with neuroticism strongly associated with increased anxiety, depression, and stress, while traits like extraversion and conscientiousness offered protective benefits. Regression analysis further emphasized pornography addiction’s negative impact, exacerbating neuroticism and reducing resilience. Gender differences were also noted, with female participants scoring higher on the BFI, suggesting gender-specific variations in how personality traits interact with addiction and mental health.

The findings underscore the need for targeted interventions, such as workshops on stress management, emotional regulation, and inclusive sexual education, to support nursing students’ well-being. Additionally, integrating awareness programs on internet addiction and its psychological impacts into nursing curricula is crucial. These programs should educate students about addiction signs, mental health effects like anxiety and depression, and strategies for managing online behavior. By incorporating modules on digital wellness and healthy screen habits, students can better mitigate risks associated with excessive internet use.

To explore causal relationships, future studies should adopt longitudinal designs to track changes in pornography use, mental health, and personality traits over time. Additionally, mixed-method approaches combining surveys with in-depth interviews could provide deeper insights into cultural and psychological factors influencing addiction, offering a more comprehensive understanding of this complex issue. Future research should also explicitly investigate gender differences in response rates and data collection challenges, particularly in culturally conservative settings.

### Nursing implications

This study underscores the need for actionable steps to address pornography addiction and its associated mental health challenges among nursing students. Institutional policies play a critical role in reducing stigma and promoting mental health awareness, particularly in culturally conservative settings like Egypt. Universities should implement anti-stigma campaigns and integrate mental health education into curricula to normalize discussions about sensitive topics like pornography addiction. Establishing confidential counseling services and peer support programs can create safe spaces for students to seek help without fear of judgment. Additionally, institutions should develop clear guidelines for addressing mental health issues and promote responsible internet use through workshops and awareness programs.

Educators should implement targeted interventions, such as workshops on stress management, emotional regulation, and inclusive sexual education while integrating mental health awareness and resilience-building into the curriculum. Policymakers must allocate funding for research and evidence-based interventions; develop guidelines for addressing these issues in educational settings, and support gender-specific approaches to account for variations in personality traits and mental health outcomes. Healthcare providers should screen for pornography addiction and mental health issues, offer counseling and therapy services, and educate students on healthy coping mechanisms.

## Data Availability

The dataset generated and/or analyzed during the current study is available from the corresponding author upon reasonable request.
